# Data on COVID-19 vaccination intention and its predictors in Poland

**DOI:** 10.1016/j.dib.2022.108227

**Published:** 2022-04-29

**Authors:** Trepanowski Radosław, Drążkowski Dariusz

**Affiliations:** Faculty of Psychology and Cognitive Science, Adam Mickiewicz University, 89 Szamarzewskiego Street, Poznan PL-60-568, Poland

**Keywords:** Theory of planned behavior, Reactance, Disease severity, Moral norms, Attitude

## Abstract

With Coronavirus disease 2019 (COVID-19) continuous spread there is a great need for understanding factors leading to vaccination in order to effectively encourage people to take the vaccine. The current dataset consists of a number of variables potentially explaining vaccination intention, that is: perceived disease severity, trait-reactance, demographics and variables associated with The Theory of Planned Behavior (attitudes toward vaccination, importance of social and moral norms when considering vaccination, perceived behavioral control). The sample consists of 551 self-reports, based on the demographic (gender and age) structure of 18–70+ year-olds in Poland. This data might be used for further exploration of the relationships between trait reactance and COVID-19 vaccination intention, as well as for cross-cultural comparisons. In the original paper, the authors have conducted a series of factor analyses, regressions, correlations and developed a structural model.

## Specifications Table

**Table:** 

Subject	Psychology
Specific subject area	Health psychology, social psychology
Type of data	Primary data, tables
How the data were acquired	Data was obtained by a Polish national research panel (Ariadna; https://panelariadna.pl/) by the method of online survey. The original survey is no longer available, thus all the items used in the study, as well as instructions are available alongside the data on the Open Science Framework (OSF; https://osf.io/t247k/)
Data format	Raw, Analyzed (complete analyses are available in the original paper [1], Filtered (descriptive statistics)
Description of data collection	Survey data was obtained from 551 Polish people (random quota sample) invited to participate, based on the demographic (gender and age) structure of 18–70+ year-olds in Poland.
Data source location	The data was collected online from respondents residing in any location in Poland.
Data accessibility	Data is deposited on the Open Science Framework. Repository name: Open Science Framework Direct URL to data: https://osf.io/t247k/
Related research article	D. Drążkowski, R. Trepanowski, Reactance and perceived disease severity as determinants of COVID-19 vaccination intention: an application of the theory of planned behavior, Psychology, Health & Medicine (2021). doi:10.1080/13548506.2021.2014060.

## Value of the Data

• This dataset allows for exploration of: (1) COVID-related variables such as perceived COVID-19 severity and the intention to vaccinate; (2) vaccination-related variables within the context of the Theory of Planned Behavior (TPB) [2], that is social norms, moral norms, attitudes, behavioral control; (3) trait-reactance.
• The data contains a number demographic variables, such as sex, age, place of residence, education level, being ever aflicted with COVID-19 and knowing someone who was/is afflicted with COVID-19.
• The data might be especially useful for cross-cultural comparisons if used with similar data from different countries, as it is based on the demographic structure for age and gender in Poland.
• This data can be statistically analyzed in order to further explore the relations between trait reactance and COVID-19.
• As most of the questionnaires used in the data collection process were designed by the authors, it is possible to further explore the relations between the items by the means of factor analyses or use those items in the design of further measures.

## 1 Data Description

The described dataset provides information on COVID-19 vaccination intention, perceived severity of COVID-19, attitudes toward vaccination, importance of social and moral norms when considering vaccination, perceived behavioral control over vaccinating and trait reactance among Polish people. 551 responses are included within the datasat. The respondents were aged 18–86 (*M* = 45.34; *SD* = 14.09). 276 (50.1%) were males, while 275 were females (49.9%). Most had either higher (34.1% or middle education (32.3%), and lived mostly in villages (35.2%), medium cities (20–99 thousand residents; 19.8%) or big cities (100–500 thousand residents; 19.2%). Overall, the data was consistent with demographic structure in Poland. Furthermore, 90.9% of respondents have not contracted COVID-19, while 9.1% have. 70.4% knew someone who had contracted COVID-19, 29.4% had not, and 0.2% had no knowledge whether they had or not.

The raw data is stored in .sav and .csv files that are available at OSF. Items 1-2 represent participants ID number and the time needed to finish the survey. Items 3–5 measured perceived severity of COVID-19, while items 6–16 measured trait reactance with the Hong Psychological Reactance Scale (HPRS) [3]. Items 17–32 represent the variables associated with the Planned Behavior Theory (social norms, moral norms, attitudes, behavioral control, intention to vaccinate against COVID-19*).* Items 33–39 provide information on participants age, sex, place of residence, education level, whether they were afflicted with COVID-19 and know someone who was or is. Item 40 is a recoded version of item 39, in such a way, that the “do not know” answer is set to “no”. Items 41–47 represent calculated scales. Detailed item descriptions are reported in Supplementary Tables 1 and 2 (available at OSF). It should be noted that two of the measures (attitude and behavioral control) use differing anchors, those are presented within the supplementary file. The remaining measures use either the completely false/true or completely agree/disagree anchors. This is also reported in detail in the codebook (supplementary files). Table 1 consists of descriptive statistics for the calculated measures, all of which were averaged. The supplementary file consists of all the items and instructions used in the survey in both Polish and English.

**Table 1:** Descriptive statistics for the calculated measures.

Measure	M	SD
*Hong Psychological Reactance Scale*	4.23	.94
*Perceived severity of COVID-19*	5.14	1.36
*COVID-19 vaccination intention*	4.28	1.77
*Attitudes towards vaccination*	4.44	1.72
*Importance of social norms for vaccination*	4.22	1.52
*Importance of moral norms for vaccination*	4.30	1.67
*Behavioral control over vaccination*	4.79	1.15

## 2 Experimental Design, Materials and Methods

The data was gathered by a Polish national research panel Ariadna. The participants were invited to take part in the study based on their demographics, so as to gather a sample representative for the demographic structure of Polish 18-70+ year-olds. Before taking part in the survey, the participants were presented with the instructions describing the aim of the survey, their rights as participants, details on the authors alongside a contact address. The information about compensation for taking part in the study (each participant received points exchangeable for rewards within the panel) was presented to the participants by Ariadna before the survey. The participants were informed that they will be taking part in two separate studies - only the data from the second one is currently reported. After that, the participants provided informed consent. Next, each participant completed a set of questionnaires in the following order: Perceived severity of COVID-19, Hong Psychological Reactance Scale, Attitudes towards vaccination, Importance of social norms for vaccination, Importance of moral norms for vaccination, COVID-19 vaccination intention. Finally the participants filled a demographic survey with questions regarding age, sex, place of residence, education level, being afflicted with COVID-19 and knowing someone afflicted with COVID-19. In the original paper [1] the data was analyzed using frequency analyses, correlation analysis, regressions, factor analysis and structural equation modeling. Measures used in the survey are described in Table 2. Beside the Hong Psychological Reactance Scale, all the measures were designed by the authors of the current paper. Measures related to the TPB were designed in accordance with pointers provided by Francis et al. [4].

**Table 2:** Measures and descriptions.

Measure	Description
*1. Hong Psychological Reactance Scale*	A scale measuring trait-reactance, that is a tendency to experience a state of reactance - motivational state that drives freedom restoration [5]. The scale consists of 11 items rated on a seven-point scale (1 = “completely false” to 7 = “completely true”). α = .90.
*2. Perceived severity of COVID-19*	This scale measures whether someone believes that COVID-19 is a severe, possibly life-threatening disease that might be dangerous to them in case of contracting it. The scale consists of three items rated on a seven-point scale (1 = “completely disagree” to 7 = “completely agree”). α = .93.
*3. COVID-19 vaccination intention*	A scale that measures intention to take the COVID-19 vaccine, when it becomes available. The scale consists of three items rated on a seven-point scale (1 = “completely false” to 7 = “ completely true”; *α* = .96).
*4. Attitudes towards vaccination*	This scale assesses perceptions of possible consequences of getting vaccinated against COVID-19. It consists of three seven-point bipolar adjective measures. *α* = .95.
*5. Importance of social norms for vaccination*	The scale measures beliefs about the opinions regarding participant's possible vaccination that others may hold. It consists of three items rated on a seven-point scale (1 = “completely disagree” to 7 = “completely agree”). α = 97.
*6. Importance of moral norms for vaccination*	This scale assesses moral responsibilities that one holds in relation to COVID-19 vaccination. The scale consists of three items rated on a seven-point scale (1 = “completely disagree” to 7 = “completely agree”). α = .96.
*7. Behavioral control over vaccination*	A scale measuring perceived control over the process of own vaccination. It consists of four items rated on seven-point bipolar scales. α = .75.

## Ethics Statements

The authors assert that all procedures contributing to this work comply with the ethical standards of the Institutional Research Committee (Ethical Committee of Faculty of Psychology and Cognitive Science, Adam Mickiewicz University in Poznan) and with the Helsinki Declaration of 1975, as revised in 2008 as well. All participants provided informed consent.

## CRediT authorship contribution statement

**Trepanowski Radosław:** Conceptualization, Methodology, Writing – original draft, Data curation, Formal analysis, Resources. **Drążkowski Dariusz:** Conceptualization, Methodology, Resources, Writing – review & editing, Funding acquisition.

## Declaration of Competing Interest

The authors declare that they have no known competing financial interests or personal relationships that could have appeared to influence the work reported in this paper.

## Data Availability

Data on COVID-19 vaccination intention and its predictors in Poland (Original data) (OSF - Open Science Framework).
